# Evaluation of cortical and trabecular bone structure of the mandible in patients using L-Thyroxine

**DOI:** 10.1186/s12903-023-03670-z

**Published:** 2023-11-20

**Authors:** Melike Gulec, Melek Tassoker, Mediha Erturk

**Affiliations:** 1 Department of Dentomaxillofacial Radiology, Karamanoğlu Mehmetbey University Faculty of Dentistry, Karaman, Turkey; 2 Department of Dentomaxillofacial Radiology, Necmettin Erbakan University Faculty of Dentistry, Bağlarbaşı sk, Meram, Konya, 42050 Turkey

**Keywords:** Hypothyroidism, Medication, Osteoporosis, Mandibular cortical index, Fractal dimension, Image processing

## Abstract

**Background:**

Long-term use of L-Thyroxine (LT4), the synthetic thyroxine hormone used for thyroid hormone replacement therapy, is an important risk factor for osteoporosis. The aim of this study was to investigate the differences between mandibular cortical index (MCI) and trabecular bone fractal dimension (FD) values on panoramic radiographs of patients using LT4 and control subjects.

**Methods:**

A total of 142 female patients, 71 cases and 71 controls, were analyzed in the study. Ages were matched in case and control groups and the mean age was 36.6 ± 8.2 (18 to 50) years. MCI consisting of C1 (Normal Mandibular Cortex), C2 (Moderately Resorbed Mandibular Cortex) and, C3 (Severely Resorbed Cortex) scores was determined for case and control groups. Fractal analysis was performed using ImageJ on selected regions of interest from the gonial and interdental regions. The box-count method was used to calculate FD values. Wilcoxon signed-rank test, Mann-Whitney U test, and Spearman correlation analysis were applied to compare the measurements. Statistical significance of differences was established at *P* < 0.05 level.

**Results:**

FD values did not show statistically significant differences between case and control groups (*p* > 0.05). The mean FD in the right gonial region was 1.38 ± 0.07 in the case group and 1.38 ± 0.08 in the control group (*p* = 0.715). The mean FD in the right interdental region was 1.37 ± 0.06 in the cases and 1.36 ± 0.06 in the control group (*p* = 0.373). The mean FD in the left gonial region was 1.39 ± 0.07 in the cases and 1.39 ± 0.07 in the control group (*p* = 0.865). The mean FD in the left interdental region is 1.37 ± 0.06 in the cases and 1.38 ± 0.05 in the control group (*p* = 0.369). The most common MCI score was C1, with 62% in the cases and 83.1% in the control group. MCI scores showed a statistically significant difference between cases and controls (*p* = 0.016, *p* < 0.05). While the C2 score was higher in the cases, the C1 score was higher in the controls.

**Conclusions:**

LT4 use was not associated with the FD of mandibular trabecular bone, but was associated with MCI values of cortical bone. Further studies on larger samples with different imaging modalities and image processing methods are needed.

## Introduction

Thyroid hormones, which are effective in maintaining anabolism-catabolism balance, are involved in many important metabolic reactions in the body. Thyroid hormones (T3, T4) are produced by stimulation of the thyroid gland through thyroid stimulating hormone (TSH) released from the pituitary gland. In case of deficiency or excess, many tissues and organs are affected. Hypothyroidism develops as a result of insufficient secretion of thyroid hormones. The most common causes of primary hypothyroidism, known as thyroid gland-induced hypothyroidism, include iodine deficiency, autoimmune thyroid disease (Hashimoto’s thyroiditis), thyroidectomy, medications, radiotherapy to the neck, congenital disorders in thyroid gland development or thyroid hormone synthesis [1].

The prevalence of primary hypothyroidism, one of the most common endocrine diseases, is between 3.8 and 4.6% in the general population and its prevalence is reported to be increasing. The prevalence increases with age. It is 5–10 times more common in women compared to men. The diagnosis is made biochemically. In cases of primary hypothyroidism, the TSH level is above the reference range while the free T4 level is low. Thyroid hormone replacement therapy has been used for more than 100 years. The daily replacement dose of L-Thyroxine (LT4), the synthetic thyroxine hormone used for this purpose, is 1.4–1.8 mcg/kg on average, and the initial dose may vary depending on the patient’s age, comorbidities, and duration of hypothyroidism. Long-term use of LT4 has been reported to increase osteoclast activation by affecting the turnover rate in bone and is an important risk factor for osteoporosis [2].

Structures that cannot be defined by standard geometric shapes and show similar properties when viewed from different scales are defined as fractals [3]. Fractal analysis is a method that has been increasingly used in recent years because it is easily accessible, is not affected by variables such as projection geometry and radiodensity, and can provide objective data on the internal trabecular structure. Fractal dimension (FD) refers to the degree of complexity that emerges by analyzing similar structures within the fractal structure. Bone morphology also has a fractal structure due to its trabeculation feature. Lower FD values for trabecular bone are associated with resorption [4].

Radiomorphometric indices such as panoramic mandibular index, mandibular cortical width, and mandibular cortical index (MCI), which are evaluated on panoramic radiographs, are related to the bone mineral density of the general skeleton and allow to examine of the signals of bone resorption and evaluate bone quality with the use of determined reference points. Among these, MCI is more practical than other methods since it does not require any measurement. The decrease in cortex width from C1 to C3 index score is associated with a decrease in bone density [5].

LT4 is the gold-standard therapeutic agent for the treatment of hypothyroidism. Long-term use of LT4 has been associated with heart disease, osteoporosis and bone fractures [6]. Bone strength depends on its mineral content and also cortical-trabecular microarchitecture. Osteoporosis can negatively affect both of these factors, leading to loss of total bone mass, reduction in cortical thickness, disruption of trabecular microarchitecture and dissolution effects on trabecular bone tissues. Fractures of the mandible or maxilla due to osteoporosis, which may also be caused by long-term LT4 use, significantly affect the quality of life of these patients. The treatment of these fractures is challenging as it requires a multidisciplinary approach [7, 8] and predicting the risk of osteoporosis is of great importance in individuals in the risk group [9]. Although it has been reported to increase the risk of fracture by decreasing bone mineral density in the forearm, hip, femoral head, and vertebrae, there is no data on its effect on the jaws in the literature. The aim of this study was to compare the MCI and mandibular trabecular bone FD values in LT4 users with healthy subjects.

## Methods

### Sample and study design

Within the scope of this study, anamnesis and panoramic radiography records of individuals who applied to the Department of Dentomaxillofacial Radiology, Faculty of Dentistry, Necmettin Erbakan University, for dental examination for various reasons between 2020 and 2023 were retrospectively analyzed. The study protocol was laid out in accordance with the principles defined in the Declaration of Helsinki, including all amendments and revisions. The study was evaluated by the Ethics Committee of the Necmettin Erbakan University and approved for compliance with ethical principles (Decision No: 2023/273).

The case group of the study consisted of 71 female patients between 18 and 50 years of age taking LT4. Only premenopausal women were included in the study to avoid the osteoporotic effect of postmenopause on bone metabolism. Seventy-one systemically healthy individuals, age-matched to the case group, also constituted the control group of the study. Women in postmenopause, individuals with other systemic conditions affecting bone metabolism (Paget’s disease, renal osteodystrophy, osteomalacia, etc.), or individuals using another therapeutic agent (bisphosphonate, etc.) that may affect bone metabolism, panoramic images with central pathologies in the mandible, and panoramic radiographs without diagnostic adequacy for fractal analysis were excluded.

### Radiographic examination

All digital panoramic images examined in the study were obtained using a 2D Veraviewpocs (J MORITA MFG Corp., Kyoto, Japan) digital panoramic X-ray machine with 70 kV, 5 mA, and 15 s irradiation time parameters.

A 3.10 GHz Intel 10th Generation i5 with 8 GB RAM, Windows 10 Professional operating system, and a 21.5-inch flat panel color screen (Lenovo ThinkVision S22e-20) with a resolution of 1920 × 1080 pixels and a resolution of 3.10 GHz was used to examine the images saved in ‘TIF’ (Tagged Image File) format.

For standardization of the images, the size of all images was set to 2836 × 1500 pixels using Adobe Photoshop CS5 (Adobe Systems Inc., San Jose, CA). For fractal analysis, ImageJ v1.41, a version of the National Institutes of Health Image software with 64-bit Java for Windows, was used. The program was downloaded from the internet at https://imagej.nih.gov/ij/download.html. 20% of the data were repeated twice at 15-day intervals by the same observer.

### Image processing: fractal analysis

**Fig. 1:**
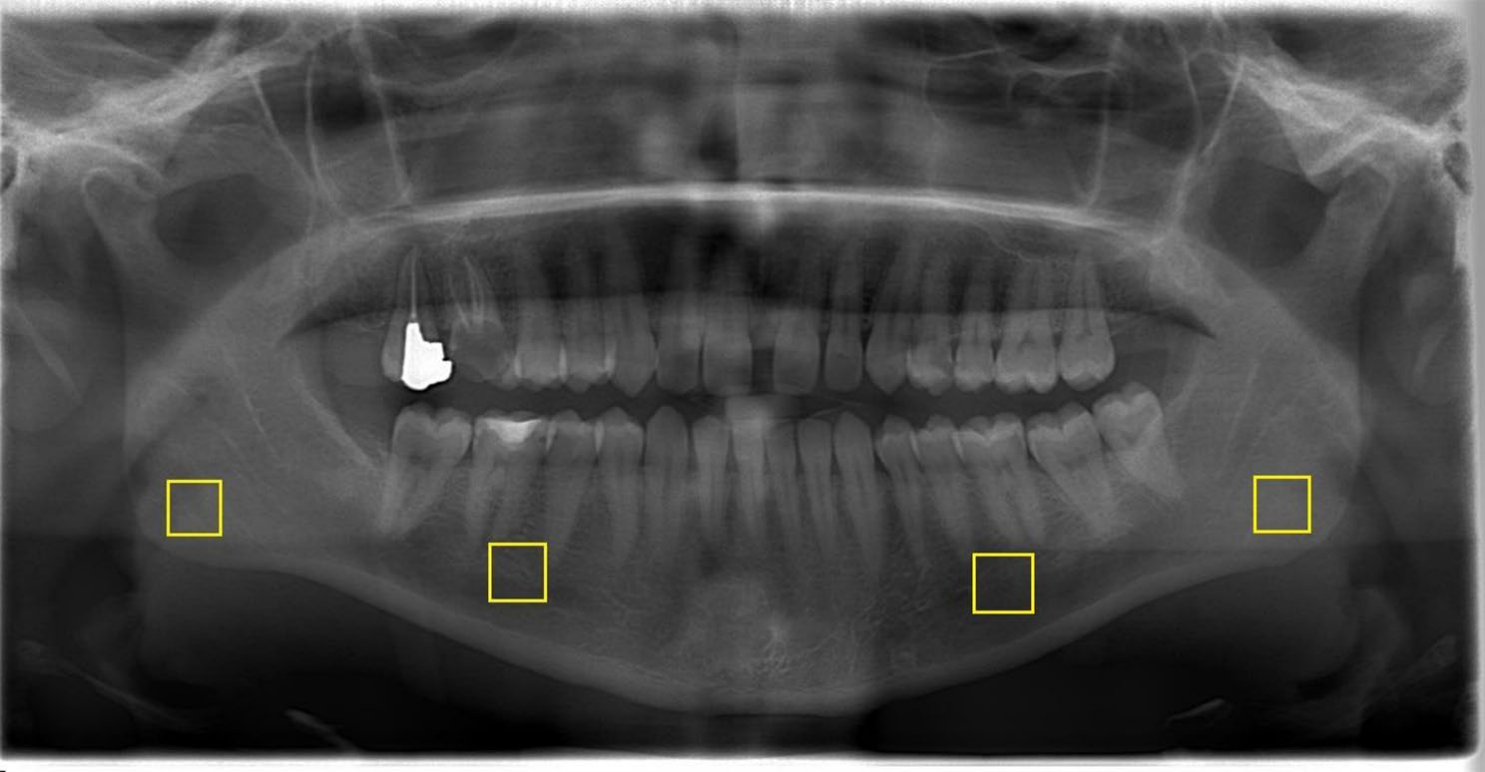
Selecting the specified ROIs on the program

On panoramic radiographs, a total of 4 regions of interest (ROI) of 100 × 100 pixels were selected. The first ROI was from the interdental regions of the right-left mandibular second premolars and first molars (excluding the periodontium and the cortical borders of the mandibular canal) and the second ROI was from the right and left mandibular angulus (Fig. 1).

**Fig. 2:**
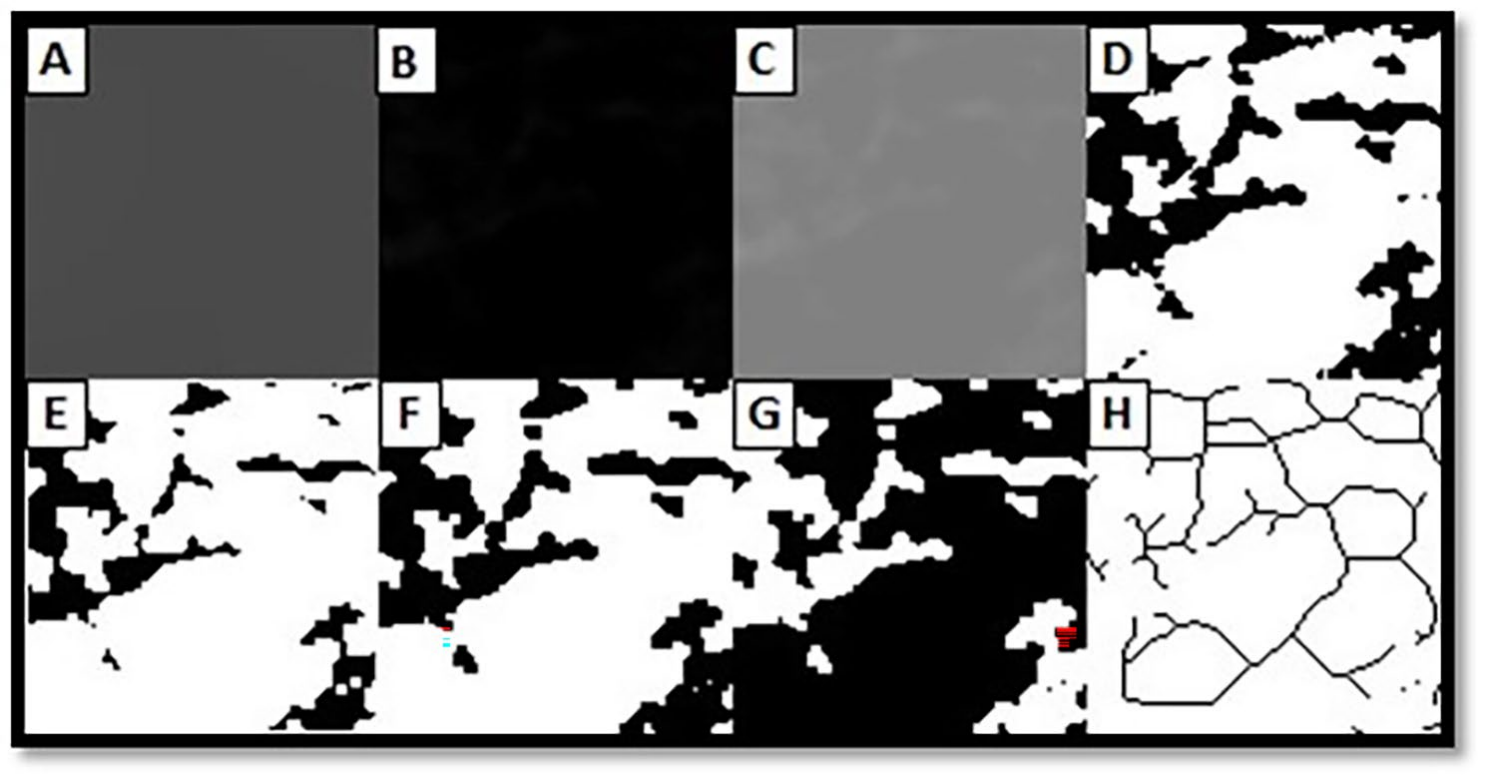
**A**, Blurring; **B**, Subtracting the blurred image from the original image; **C**, Adding 128 shades of gray; **D**, Converting to black-and-white image; **E**, Reducing noise with Erode; **F**, Expanding with Dilate; **G**, Inverting colors; **H**, Converting to skeletal format

The box-count method described by White and Rudolph [10] was used to calculate FD values According to this method;

- First, the copied ROIs are blurred using the ‘Gaussian Blur’ filter (sigma, 35 pixels) (Fig. 2A). The blurred processed image is subtracted from the original first image (Fig. 2B) and 128 Gy values for each pixel are added to the image (Fig. 2C).
- With the ‘Make Binary’ option, the image is converted to a two-color format in black and white (Fig. 2D).
- The ‘Erode’ step is applied to reduce the noise on the image (Fig. 2E).
- With the ‘Dilate’ option, the existing areas are enlarged and made more prominent (Fig. 2F).
- In the ‘Invert’ step, white areas on the image are converted to black and black areas to white to reveal the outline of the trabecular bone (Fig. 2G).
- Finally, with the ‘Skeletonize’ option, the image in which the trabecular structure is skeletally determined is made ready for fractal analysis (Fig. 2H). The ‘Fractal box counter’ command under the ‘Analyze’ button is used to calculate the FD.

### Mandibular cortical index (MCI)

**Fig. 3:**
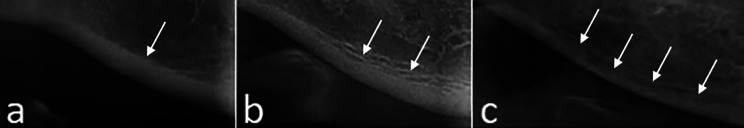
MCI description on cropped panoramic images (**a**:C1, **b**:C2, **c**:C3)

In MCI defined by Klemetti et al. [11] bone resorption in the cortical region extending from the distal foramen mentale to the antegonial region is examined. According to this index:

- C1 (Normal Mandibular Cortex): The margins are equal and sharp on both sides of the cortex (Fig. 3a).
- C2 (Moderately Resorbed Mandibular Cortex): The endosteal margins of the cortex show half-moon-shaped defects (lacunar resorption) and the margins are observed as 1–3 layers (Fig. 3b).
- C3 (Severely Resorbed Cortex): Cortical cortices are severely porous and dense endosteal debris is present (Fig. 3c).

In determining the final MCI index, the right, and left sides are scored, and the class with more morphologic destruction is preferred to the class with less destruction.

### Statistical analysis

Data were analyzed using SPSS v21.0 (IBM Corp, Armonk, NY, USA). Descriptive statistics were calculated for all parameters in the study. FD measurements and MCI examination were repeated twice at 14-day intervals for 25% of the data by the same oral radiologist. The second measurements were completely blinded to the first measurements. Intraclass correlation coefficient (ICC) and Kappa analysis were applied to assess intraobserver agreement. Wilcoxon signed-rank test, Mann-Whitney U test, and Spearman correlation analysis were applied to compare the measurements. Chi-square analysis was applied to determine the relationships between categorical variables. A value of *p* < 0.05 was considered statistically significant in all analyses.

## Results

A total of 142 female patients, 71 cases and 71 controls, were analyzed in the study. Ages were matched in case and control groups and the mean age was 36.6 ± 8.2 (18–50) years. The dose of LT4 use of the patients varied between 25 and 200 mcg daily and their distribution is given in Table 1. The most frequently used dose was 100 mcg/day (39.4%), followed by 125 mcg/day (15.5%), 75 mcg/day (12.7%) and 50 mcg/day (12.7%). The least frequent doses are 175 mcg/day (1.4%) and 200 mcg/day (1.4%).

**Table 1:** Patients’ daily doses of LT4

LT4 (mcg/day)	Number of patients	%
25	6	8,5
50	9	12,7
75	9	12,7
100	28	39,4
125	11	15,5
150	6	8,5
175	1	1,4
200	1	1,4
Total	71	100,0

There was no statistically significant difference in FD values between the case and control groups (*p* > 0.05). The mean FD values calculated from the gonial and interdental regions are shown in Table 2. The mean FD in the right gonial region was 1.38 ± 0.07 in the case group and 1.38 ± 0.08 in the control group (*p* = 0.715). The mean FD in the right interdental region was 1.37 ± 0.06 in the case group and 1.36 ± 0.06 in the control group (*p* = 0.373). The mean FD in the left gonial region was 1.39 ± 0.07 in the case group and 1.39 ± 0.07 in the control group (*p* = 0.865). The mean FD in the left interdental region is 1.37 ± 0.06 in the case group and 1.38 ± 0.05 in the control group (*p* = 0.369). The mean FD measurements in the left gonial region are at the highest values, and the mean FD measurements in the right interdental region are at the lowest values (Table 2). Based on the Spearman analysis, there was no significant correlation between the daily dose of LT4 and FD values (*p* > 0.05, r=-0.078-0.175). The intraobserver agreement for FD measurement was good (ICC = 0.72).

**Table 2:** Mean FD values in the different ROIs measured

ROI		Number of patients	FD	Mann-Whitney U
right_gonial	case	71	1,38 ± 0.07	*p* = 0,715
	control	71	1,38 ± 0.08	
right_interdenal	case	71	1,37 ± 0.06	*p* = 0,373
	control	71	1,36 ± 0.06	
left_gonial	case	71	1,39 ± 0.07	*p* = 0,865
	control	71	1,39 ± 0.07	
left_interdental	case	71	1,37 ± 0.06	*p* = 0,369
	control	71	1,38 ± 0.05	

In the calculations performed without making a case-control distinction; when the right and left measurement values were compared, it was observed that the interdental and gonial regions did not differ significantly from each other (*p* > 0.05). While the right interdental mean FD value is 1.36 ± 0.06, it is 1.37 ± 0.06 on the left. While the average FD value in the right gonial is 1.38 ± 0.08, it is 1.39 ± 0.07 in the left. In the examinations performed on the same side, the FD values of the right gonial and right interdental region showed a statistically significant difference from each other (*p* = 0.022, *p* < 0.05). Left gonial and left interdental FD values were significantly different from each other (*p* = 0.039, *p* < 0,05). Gonial region mean FD values (1.38 on the right and 1.39 on the left side) were higher than interdental region FD (1.36 on the right and 1.37 on the left side) values (Table 3).

**Table 3:** FD values of all patients

ROI	Number of patients	FD
right_gonial	142	1,38 ± 0.08
right_interdental	142	1,36 ± 0.06
left_gonial	142	1,39 ± 0.07
left_interdental	142	1,37 ± 0.06

When the MCI distribution was examined, the most common score was C1, with 62% in the case group and 83.1% in the control group. MCI scores showed a statistically significant difference between case-control groups (*p* = 0.016, *p* < 0.05). Intraobserver agreement for MCI evaluation was excellent (Kappa = 0.87). While the C2 (Moderately Resorbed Mandibular Cortex) score was higher (36.6% of the patients) in the case group, the C1 score (Normal Mandibular Cortex) was higher (83.1% of the patients) in the control group (Table 4).

**Table 4:** Distribution of MCI scores in case-control group

		MCI			Total	*χ²*
		C1	C2	C3		
Case	Number of patients	44	26	1	71	
	%	(62%)	(36,6%)	(1,4%)	(100%)	
Control	Number of patients	59	11	1	71	*p = 0,016*
	%	(83,1%)	(15,5%)	(1,4%)	(100%)	
Total	103	37	2	142		
%	(72,5%)	(26,1%)	(1,4%)	(100%)		

Among the 71 patients in the case group, the most frequently used dose was 100 mcg/day (n = 28), the least used doses were 175 mcg/day (n = 1) and 200 mcg/day (n = 1). There was no statistically significant relationship between the daily dose of LT4 and MCI scores (*p* = 0.426, *p* > 0.05) (Table 5).

**Table 5:** Distribution of MCI scores according to different LT4 doses

			MCI			Total	*χ²*
			C1	C2	C3		
Doses (mcg)	25	Number of patients	4	2	0	6	
		%	(66,7%)	(33,3%)	(0%)	(100%)	
	50	Number of patients	8	1	0	9	
		%	(88,9%)	(11,1%)	(0%)	(100%)	
	75	Number of patients	4	4	1	9	
		%	(44,4%)	(44,4%)	(11,1%)	(100%)	
	100	Number of patients	16	12	0	28	
		%	(57,1%)	(42,9%)	(0%)	(100%)	
	125	Number of patients	5	6	0	11	*p = 0,426*
		%	(45,5%)	(54,5%)	(0%)	(100%)	
	150	Number of patients	5	1	0	6	
		%	(83,3%)	(16,7%)	(0%)	(100%)	
	175	Number of patients	1	0	0	1	
		%	(100%)	(0%)	(0%)	(100%)	
	200	Number of patients	1	0	0	1	
		%	(100%)	(0%)	(0%)	(100%)	
Total		Number of patients	44	26	1	71	
		%	(62%)	(36,6%)	(1,4%)	(100%)	

## Discussion

Thyroid hormones have important roles in both growth and development and homeostasis. Hypothyroidism, defined as thyroid hormone insufficiency and rarely ineffectiveness at the tissue level, is up to 10 times more common in women than in men. Synthetic thyroid hormone LT4 is routinely used for thyroid hormone replacement [12]. Dentists frequently encounter patients using LT4 in their daily treatment routine. However, to our knowledge, there is no study in the literature examining the effect of LT4 on the jaw bones. Since it has been reported in previous studies that osteopenic and osteoporotic changes in bone have been observed in long-term use of LT4 above the replacement dose [6] the dose of LT4 used in this study was also recorded.

Thyroid hormones are responsible for bone growth and development at physiologic levels. Studies have reported that the balance between bone formation and resorption is disrupted in postmenopausal women due to decreasing estrogen levels in the postmenopausal period and the risk of osteopenia and osteoporosis increases [13]. Since it is assumed that there is no or minimal loss of bone mass in the premenopausal period [14], only premenopausal women were included in the study among LT4 users. Since hypothyroidism is 5–10 times more common in women [15] and accordingly, individuals using LT4 are mostly women and FD may be different in men and women [16], the study was performed only with women. Mineral density in the jaw bones is in line with the mineral density of other bones in the body [17]. One of the areas most affected by resorption in the jaws is the mandibular posterior region [18, 19]. This study investigated whether LT4 causes differences in trabecular bone and mandibular cortex in the mandibular posterior region.

Bone mineral density (BMD) measurements, which are used as the gold standard in the evaluation of osteoporosis, are generally difficult to access, time-consuming, invasive, and expensive [20]. The fractal analysis method, which has been used as an alternative to BMD measurements in recent years and evaluates trabecular bone morphology, is a method that is increasingly being used in medicine and dentistry due to its ease of use and access, non-invasiveness, projection angle up to 20°, not being affected by variables such as radiation dose and providing objective data, although it has certain limitations. Since the most commonly used method for fractal analysis is the box-counting method, this method was preferred in this study. In this study, trabecular bone FD values calculated from the mandibular gonial and interdental regions on panoramic radiographs of premenopausal women who received different doses of LT4 were analyzed in comparison with the control group individuals. It has been reported that dental structures should not be included within the ROI boundaries in trabecular bone FD studies [21]. In the present study, fractal analysis was applied to ROIs determined as 100 × 100 pixels in gonial regions without cortical borders, and 100 × 100 pixels in interdental regions without periodontium and mandibular canal cortical borders.

The findings of studies examining the relationship between long-term LT4 use and BMD measurements are contradictory. Mendonça et al. [22] performed BMD measurements with dual-energy X-ray absorptiometry (DEXA) in their study on 17 thyroid cancer and 34 healthy control subjects who underwent total thyroidectomy and received LT4 therapy for at least 5 years and found that there was no significant relationship between long-term LT4 use and BMD. On the other hand, Bin-Hong et al. [23] performed BMD measurements on 65 thyroid cancer women who received LT4 therapy for 1.5 to 9 years, and 50 control subjects, all premenopausal women. As a result, they found that BMD measurements were lower in the study group and stated that long-term LT4 therapy increases the turnover rate in bone and constitutes a risk factor for decreased bone strength. In the present study, no significant correlation was found between the daily dose of LT4 use and mandibular FD values, and it can be said that LT4 use at therapeutic doses does not affect the trabecular FD values in the mandibular gonial and inderdental regions.

When the FD values of the individuals examined in this study, no significant difference was found between the right and left side measurements, while the FD values calculated from the gonial region were found to be higher than the FD values calculated from the interdental regions in the same side measurements. A high FD is associated with the complexity of the structure, while a low FD is explained by the simpler internal layout of the structure [21]. The lower FD values obtained in the interdental regions are explained by the fact that the trabecular bone in this region has a more regular trabecular arrangement to resist occlusal forces [24].

In recent years, the effects of vitamin D deficiency [25], antiepileptic drugs (AED) [16], selective serotonin reuptake inhibitors (SSRI) [26], bisphosphonates [27], systemic glucocorticoids [28], proton pump inhibitors (PPI) [29] on FD of the jaws have been investigated in the literature. Zihni Korkmaz et al. [25] reported that trabecular bone FD was lower in vitamin D deficiency. Tumani Üstdal et al. [16] conducted a study with 132 individuals, 66 AED users, and 66 controls. As a result of the study, it was reported that FD was lower in AED users and MCI was significantly different between case and control, and AED may have a resorptive effect on the jaws. However, it was reported that FD did not differ between case-control groups in one of the 3 different ROIs investigated in the male gender. Coşgunarslan et al. [26] investigated the effect of SSRI on a total of 212 individuals (106 cases and 106 controls) and reported that there was no difference in the FD values of the case and control groups in one of the 3 ROIs selected. In another study, Demiralp et al. [27] investigated the effect of bisphosphonates on mandibular FD in a total of 66 patients (33 cases and 33 controls) and reported that there was no statistically significant difference between these two groups. Kaya and Koç [29] who investigated the effect of PPI use on mandibular FD with a total of 128 individuals (64 cases and 64 controls), showed that there was no difference between the case and control groups in measurements made from 9 different ROIs and mandibular cortical width (MCW) index. Ersu et al. [28] performed an analysis using 4 different ROIs (3 trabecular, 1 cortical bone) from a total of 192 patients (96 cases-96 controls) to investigate the relationship between the use of systemic glucocorticoids and mandibular FD values. While there was no difference between case-control in 3 trabecular bone ROIs selected from the same regions as in our study, they found that FD in the 4th ROI selected from the basal cortex was statistically significantly lower than the control group. Based on this, they stated that systemic glucocorticoids may be effective on cortical bone but not on trabecular bone. While there was no difference between the case-control groups in the 2 different trabecular bone ROIs selected in this study, the difference seen in the MCI scores related to the cortex can be interpreted as LT4 affecting the mandibular cortical layer. Investigation of this result in future studies with prospective study design will provide more information.

In this study, it was investigated whether panoramic radiographs, which are frequently used in dental radiology, can be used as a screening tool to evaluate the risk of osteoporosis in individuals using LT4, which has a high prevalence in the population. There are many studies in the literature comparing BMD values with mandibular radiomorphometric measurements made on panoramic radiographs. In MCI measurements, which are among these mandibular indices, the part of the mandibular cortex distal to the mental foramen is evaluated, and it has been reported that MCI can be used to distinguish osteoporotic individuals from normal individuals [20, 30]. Knezovic et al. [31] found MCI useful in distinguishing age-related osteoporotic changes in the mandibular cortex. In addition, Zlataric and Celebic [32] found that the mandibular inferior cortical layer had a more porous structure in individuals with lower BMD values. Youssif and Elshall [33] evaluated the relationship between panoramic mandibular indexes and osteoprosis in their research on 40 postmenopausal women aged 45–65, who had previously been diagnosed with osteoporosis by DEXA. It has been stated that MCI is one of the most frequently used qualitative measurements in panoramic radiographs because, unlike other panoramic indices, it does not require determining the exact location of anatomical landmarks. High MCI scores (C2, C3) has been associated with individuals being more osteoprotic or having lower BMD, and therefore MCI has been reported to be useful in evaluating osteoporosis. On the other hand, Cakur et al. [34] reported that MCI scores do not correlate with DEXA values calculated from other bones and cannot be used as an indicator of skeletal status. Nakamato et al. [20] reported that dental practioners would tend to choose C3 cortex rather than normal cortex on panoramic radiography in their clinical practice to identify low BMD, because C3 cortexis easy to diagnose with very thin cortex or severe erosion. It is a disadvantage that the preservation of MCI values depends on experience. In this study, MCI values were evaluated by a dentomaxillofacial radiologist who is an expert in the field (8 years). In current study, it was found that the C2 score was higher in the case group and the C1 score, defined as a normal mandibular cortex, was seen at a higher frequency in the control group (*p* < 0.05). This suggests that the use of LT4 may affect the mandibular cortex in the direction of resorption and it may be recommended to investigate this situation with prospective studies.

The cross-sectional design of our study is the most important limitation. Individuals’ life habits such as nutrition (especially calcium and vitamin D deficiencies), sports habits, and caffeine and cigarette-alcohol consumption may affect bone health. Even passive cigarette exposure has been reported to lead to decreased BMD [7, 35]. Therefore, prospective studies in which such individual differences are recorded are needed.

## Conclusion

There was no correlation between LT4 use and FD of trabecular bone, but MCI scores of cortical bone showed differences. Resorptive changes may be observed in the mandibular basal cortical layer in LT4 users. Therefore, the cortical bone in LT4 users should be carefully examined by the dentist and the patient may need to be referred if changes related to osteoporosis are detected.

## Data Availability

The datasets generated and analyzed during the current study are available from the corresponding author on reasonable request.
